# Exploration of enhanced recovery after surgery in female pelvic floor reconstruction: a retrospective study

**DOI:** 10.3389/fmed.2025.1659074

**Published:** 2025-11-03

**Authors:** Ya Xiao, Shasha Hong, Yuting Wan, Jianming Tang, Jie Min, Ming Hu, Suting Li, Linxiang Chen, Li Hong

**Affiliations:** ^1^Department of Gynecology and Obstetrics, Renmin Hospital of Wuhan University, Wuhan, Hubei, China; ^2^Department of Gynecology and Obstetrics, Wuhan Jiangxia District Hospital of Traditional Chinese Medicine, Wuhan, Hubei, China

**Keywords:** pelvic organ prolapse (POP), enhanced recovery after surgery (ERAS), pelvic floor reconstruction surgery, length of hospital stay (LOS), postoperative complications

## Abstract

**Background:**

Pelvic organ prolapse (POP) is a prevalent condition treated with pelvic floor reconstruction surgery, which can be associated with significant postoperative morbidity. This study evaluated the safety and feasibility of an enhanced recovery after surgery (ERAS) protocol specifically for this procedure.

**Methods:**

A retrospective analysis was conducted on 80 patients undergoing POP surgery between March 2022 and March 2023; 39 were managed with the ERAS protocol and 41 received conventional care. The ERAS pathway comprised multimodal interventions across preoperative, intraoperative, and postoperative phases. Primary outcomes were postoperative length of stay (LOS) and hospitalization costs.

**Results:**

The ERAS group had a significantly shorter postoperative LOS (112.14 vs. 154.87 h, *p* < 0.001) and lower hospitalization costs (¥40,483.02 vs. ¥42,942.12, *p* = 0.037) than the control group. There were no 30-day readmissions or reoperations in either group. The incidence of PONV was lower in the ERAS group (2.6% vs. 19.5%, *p* = 0.029). Time to first flatus, first ambulation, full unassisted ambulation, and return to basic activities of daily living (ADLs) were all significantly shorter in the ERAS group (all *p* < 0.05), and the overall complication rate was lower. Both groups showed comparable and significant improvements in POP-Q stage and Patient Global Impression of Improvement (PGI-I) scores at 30-day follow-up, with no significant differences in VAS or Quality of Recovery-15 (QoR-15) scores between groups.

**Conclusion:**

The implementation of the ERAS protocol for pelvic floor reconstruction is safe, feasible, and effective, leading to accelerated recovery, shortened hospital stay, and reduced cost, without compromising patient safety or satisfaction. These findings support the broader adoption of ERAS in POP surgery.

## Introduction

Pelvic organ prolapse (POP) is characterized by the abnormal descent of pelvic organs due to defects in the pelvic floor muscles and connective tissues, leading to varying degrees of urinary, defecatory, and sexual dysfunction that significantly impair quality of life (1). POP is highly prevalent, with an estimated lifetime risk of undergoing surgery for POP being about 19% in women (2). Based on projections from U.S. population studies, the prevalence of POP is expected to increase by approximately 50% by 2050 (3). This high prevalence establishes POP as a major global health concern. With the aging of the global population, the incidence among older women is anticipated to rise, further intensifying associated medical and socioeconomic burdens (4). Surgery remains the primary treatment for POP. Pelvic floor reconstruction surgery is now widely used for managing severe and recurrent POP, offering advantages over traditional repair techniques, such as improved anatomical restoration, functional recovery, and long-term efficacy (5). However, patients undergoing these procedures are often elderly with multiple comorbidities. Therefore, minimizing surgery-related complications is crucial to reducing perioperative risks, decreasing recurrence rates, and optimizing surgical outcomes in this population. Additionally, the considerable hospitalization costs associated with pelvic floor reconstruction pose a significant financial burden on patients, underscoring the need for strategies that reduce complications and lower costs while adhering to patient-centered care principles (6).

The enhanced recovery after surgery (ERAS) protocol, first proposed by Danish surgeon Kehlet in 2001, comprises a series of evidence-based perioperative care measures designed to attenuate the surgical stress response and accelerate recovery (7). The concept was introduced to China by Professor Li in 2007 (8). That same year, his team published pioneering findings demonstrating the safety and efficacy of ERAS in patients undergoing gastric cancer surgery (9). Internationally, ERAS has been extensively implemented in gynecological surgery, with substantial evidence supporting its benefits, including reduced postoperative complications, shorter hospital stays, and enhanced recovery (10–12). Previous studies have confirmed the feasibility and safety of ERAS pathways in gynecological oncology and benign surgeries (13). However, reports on the application of ERAS specifically in pelvic floor reconstruction surgery remain scarce in China, indicating a gap that requires further clinical validation.

In this retrospective study, we analyzed the clinical data of 80 patients with POP who underwent pelvic floor reconstruction surgery at the Department of Gynecology and Obstetrics, Renmin Hospital of Wuhan University, between March 2022 and March 2023. Our aim was to evaluate the effect of the ERAS protocols on postoperative recovery outcomes and to contribute evidence supporting the development of a standardized ERAS pathway tailored to POP patients undergoing pelvic floor reconstruction.

## Methods

### Patient recruitment

The research protocol was reviewed by the Clinical Research Ethics Committee of Renmin Hospital of Wuhan University (Clinical Trial Number: WDRY2022-K045) and registered in the China Clinical Trial Registration Center^1^ before implementation and written informed consent was obtained from each patient. This study collected the clinical data of 80 patients with POP who underwent pelvic floor reconstruction surgery in the Department of Gynecology and Obstetrics of Renmin Hospital of Wuhan University from March 2022 to March 2023, including 39 patients accepting accelerated enhanced recovery after surgery treatment (ERAS group) and 41 patients accepting routine surgical treatment (Control group). The sample size was determined by the total number of eligible cases available during this specific study period, which is comparable to that of previous exploratory studies investigating ERAS protocols in gynecological surgery. Although *a priori* power calculation was not performed due to the retrospective nature of this study, the cohort included 39 patients managed under a structured ERAS protocol and 41 patients receiving conventional perioperative care, providing sufficient data for an initial evaluation of the protocol’s feasibility and primary outcomes. The inclusion criteria were as follows: 1. severe pelvic organ prolapse, conservative treatment is ineffective and pelvic floor reconstruction surgery is proposed; 2. ASA grade I-II; 3. No clear contraindication to laparoscopic surgery; 4. Signed a written informed consent.

Exclusion criteria: 1. Combined with gynecological malignancies; 2. Acute infection period, mental illness and other surgical contraindications; 3. Contraindications to NSAIDs (renal insufficiency, peptic ulcer, history of NSAIDs allergy, history of aspirin asthma); 4. Refusal to sign the written informed consent. A thorough review of medical records confirmed that no eligible patients meeting the inclusion criteria required exclusion due to perioperative complications or loss to follow-up before the 30-day assessment, resulting in the final cohort of 80 patients (39 in the ERAS group and 41 in the control group).

The patients in the control group received the standard gynecological care program, and the patients in the ERAS group received the gynecological ERAS program. The primary outcomes were postoperative length of stay and total hospitalization costs. The secondary outcomes were 30-day readmission rate, postoperative complications, PONV incidence, and VAS pain scores. All patients were re-examined on the 30th day after discharge.

### Study design and participants

**Figure 1:**
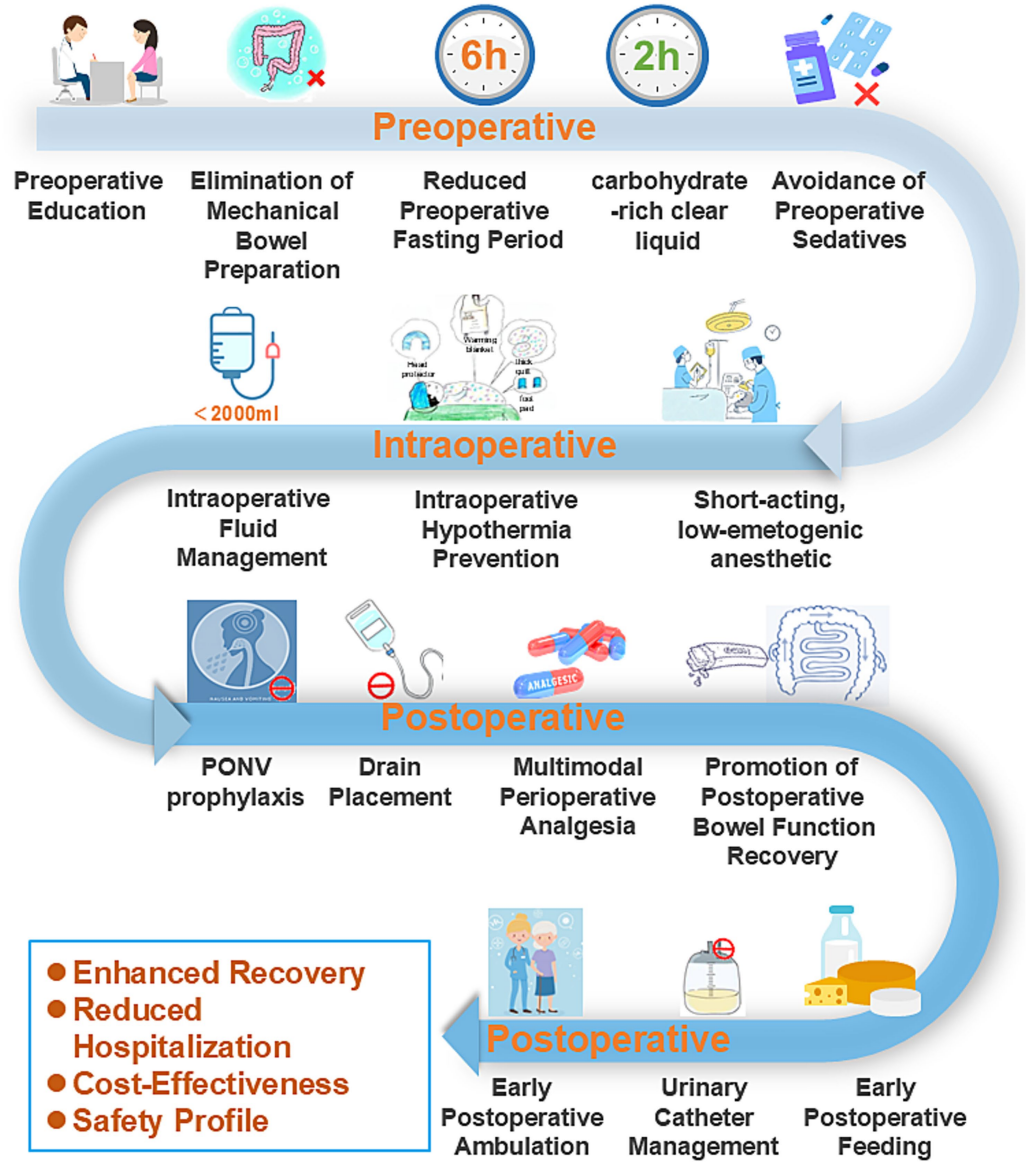
Schematic workflow of the implemented ERAS pathway.

**Figure 2:**
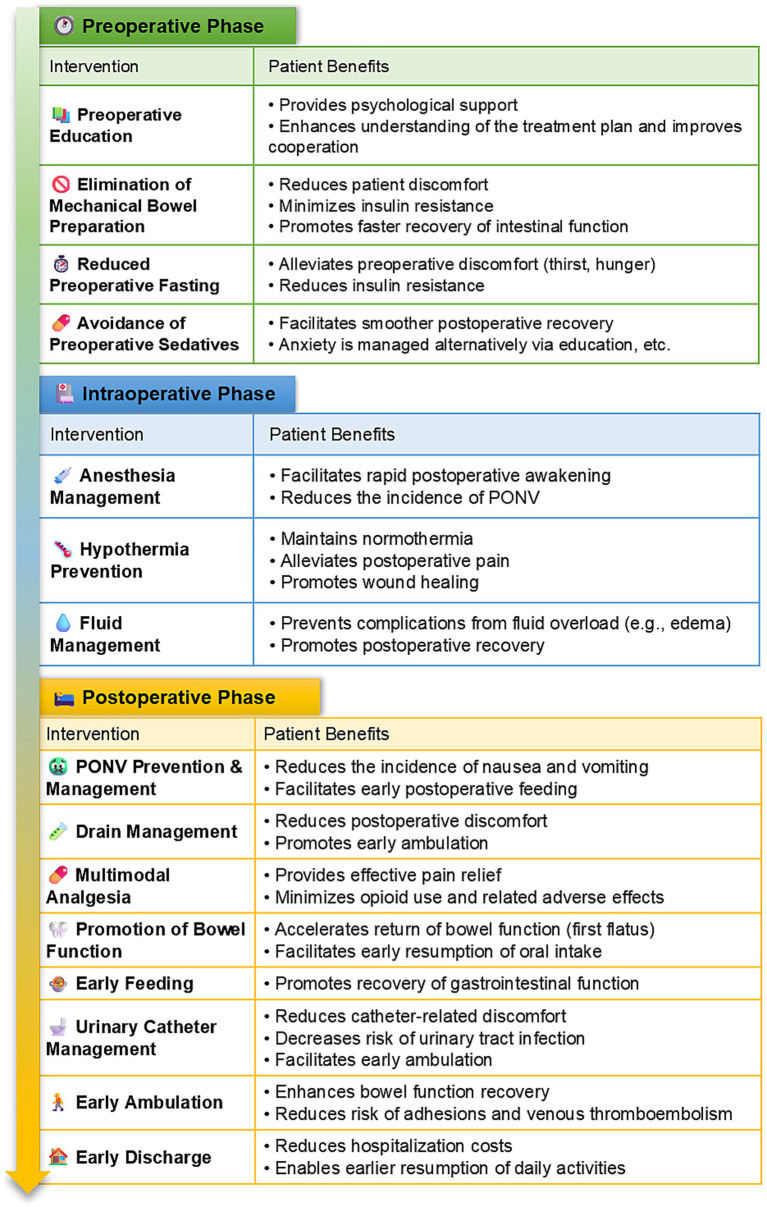
ERAS protocol interventions and benefits, by phase.

Given the absence of an established ERAS protocol specifically for urogynecological procedures, the authors developed a comprehensive ERAS protocol by integrating evidence-based practices from colorectal surgery, gynecological surgery, and their clinical expertise (14, 15). All surgical procedures were performed by the same dedicated gynecological surgical team, which ensured consistency in surgical technique and perioperative management throughout the study period. To ensure standardization, a detailed written protocol (as summarized in Table 1) was distributed to all involved healthcare staff, including surgeons, anesthesiologists, and ward nurses. A dedicated briefing session was conducted prior to study initiation to ensure consistent understanding and application. Compliance with key ERAS elements was actively monitored by the research nursing team through a standardized checklist integrated into the patient’s medical record. Compliance with the key ERAS elements was excellent, exceeding 95% across the cohort. In contrast, the control group received conventional perioperative care following standard institutional protocols. Details about the specific implementation of ERAS are shown in Figures 1, 2 and Table 1. In brief, our ERAS program for patients with POP consists of three components: preoperative, intraoperative, and postoperative interventions. Key measures that distinguished the ERAS group from the control group preoperatively included: no mechanical bowel preparation, shorter durations of preoperative fasting and fluid deprivation, and avoidance of sedative drugs. Key intraoperative measures included: the use of short-acting, low-emetogenic anesthetic drugs, the application of active warming measures for surgeries exceeding 30 min, and the restriction of intraoperative fluid intake to within 2,000 mL. The postoperative ERAS protocol was characterized by several key measures: the implementation of multimodal antiemetic therapy, avoidance of nasogastric tube placement, minimization of drain usage, NSAID-based multimodal analgesia, postoperative gum chewing to enhance gastrointestinal motility, early initiation of oral feeding, reduced duration of indwelling urinary catheterization, promotion of early ambulation, and facilitation of earlier hospital discharge based on standardized criteria.

**Table 1:** Enhanced recovery protocol for pelvic floor reconstruction surgery.

Phase	Intervention	Control group	ERAS group
Preoperative	Preoperative education	Routine preoperative medical knowledge education	Comprehensive counseling on the ERAS pathway, including recovery timeline and recommendations for early feeding/mobilization.
	Bowel preparation	Mechanical bowel preparation (enema)	No mechanical bowel preparation.
	Preoperative Fasting (h)	12 (solids)	6 (solids).
	Preoperative clear fluids (h)	4	2 (with 200 mL of carbohydrate-rich liquid 2 h before surgery).
Intraoperative	Intraoperative fluid management	According to conventional requirements	Goal-directed therapy, strictly limited to <2,000 mL
	Intraoperative warming	Routine care	Active warming measures (e.g., pre-warmed fluids, forced-air blankets).
	Multimodal analgesia/antiemetic prophylaxis	Not standardized	Multimodal analgesia: Loxoprofen Sodium 60 mg q8h, Acetaminophen 650 mg q8h, Gabapentin 150 mg (300 mg at bedtime). Antiemetic prophylaxis: e.g., Dexamethasone and/or Ondansetron.
Postoperative	Postoperative fasting	Fasting until 1 day after surgery	Water upon awakening; liquid/semi-liquid diet within 6 h after surgery.
	Multimodal pain management	On-demand	Continuation of the preoperative oral multimodal analgesic regimen.
	Promotion of bowel function	None	Chewing gum started 2 h postoperatively.
	Urinary catheter	Removed 1–3 days after surgery	Removed within 24 h after surgery.
	Mobilization	Bed rest for 1–2 days after surgery	Bed exercises within 6 h; ambulation within 12 h postoperatively.
	Discharge criteria	Standardized criteria applied: tolerance of oral intake, adequate pain control with oral analgesics, independent ambulation, spontaneous voiding, and absence of complication signs.	Standardized criteria applied: tolerance of oral intake, adequate pain control with oral analgesics, independent ambulation, spontaneous voiding, and absence of complication signs.

Visual Analogue Scale (VAS, 0–10 scale) scores were used to assess patients’ pain at 2 h, 12 h, 24 h, and 48 h postoperatively, with 1–3 being mild, 4–6 being moderate, and 7–9 being severe. The Quality of Postoperative Rehabilitation Rating Scale (QoR-15) was used to assess patients’ quality of life on postoperative days 3, 7, and 30, respectively. The patient global impression of improvement (PGI-I) score (16) was used to assess the degree of goal attainment after treatment at the postoperative 30 outpatient follow-up, and was classified into 7 levels, very favorable is 7, favorable is 6, slightly favorable is 5, no change is 4, slightly poor is 3, poor is 2, and very poor is 1.

### Statistical analysis

SPSS 26.0 software (SPSS Inc., Chicago, IL, USA) was used for statistical analysis. The Shapiro–Wilk test was used to determine the normality of the distribution of continuous variables. Student’s t test and Mann–Whitney test were used to analyze the normal distribution data and non-normal distribution data, respectively. Categorical variables were assessed by chi-square test or Fisher’s exact test. *p* < 0.05 indicated a statistically significant difference.

## Results

### Patient sociodemographic and clinical characteristics

A total of 80 patients diagnosed with pelvic organ prolapse (POP) who underwent pelvic floor reconstruction surgery were enrolled in this study. All patients completed a 30-day outpatient follow-up. The final analysis included 39 patients in the ERAS group and 41 in the control group. Preoperative systematic assessment showed no statistically significant differences between the two groups in baseline characteristics, including age, body mass index (BMI), duration of prolapse, parity, or pelvic organ prolapse quantification (POP-Q) scores (Tables 2, 3).

**Table 2:** The preoperative general conditions in two groups ($\bar{x}$ *±* s)/[M(Q1, Q3*)*].

Group/statistic	Age (year)	BMI (kg/m^2^)	Years of menopause (year)	Number of deliveries (times)
ERAS (*n* = 39)	60.87 ± 11.160	22.38 ± 1.843	14 (2, 20)	2 (1, 2)
Control (*n* = 41)	59.12 ± 11.703	23.11 ± 1.984	12 (1, 18)	2 (1, 2)
*t*-value	0.684	−1.696	−0.499	−0.010
*P*-value	0.496	0.094	0.618	0.992
Effect size (95% CI)	1.75 (−3.35, 6.85)	−0.72713 (−1.581, −1.579)	–	–

**Table 3:** POP-Q scores between the two groups before surgery [M(Q1, Q3)].

POP-Q	ERAS (*n* = 39)	Control (*n* = 41)	*P*
Aa	+0.5 (0, +1.50)	+1.0 (−0.25, +2.00)	0.527
Ba	+1.5 (+0.125, +2.00)	+1.75 (0.00, +2.50)	0.299
C	1.5 (−0.75, +2.00)	+0.50 (−1.25, +3.25)	0.985
TVL	7.00 (7.00, 7.00)	7.00 (6.00, 7.00)	0.229
Ap	−2.00 (−2.00, −1.00)	−2.00 (−2.63, 0.00)	0.619
Bp	−1.00 (−2.00, −0.5)	−1.00 (−2.00, 0.00)	0.760
D	−3.00 (−3.38, −1.13)	−3.00 (−5.00, −0.75)	0.468

### Primary outcome measures

The ERAS group had a significantly shorter postoperative hospital stay compared to the control group (112.14 ± 30.33 h vs. 154.87 ± 33.90 h; *p* < 0.001). Additionally, the total hospitalization cost was significantly lower in the ERAS group than in the control group (¥40,483.02 ± 5,441.85 vs. ¥42,942.12 ± 4,940.36; *p* = 0.037), indicating that the ERAS protocol effectively reduced medical expenses (Table 4).

**Table 4:** Primary outcomes and secondary outcomes between ERAS group and control group.

Measures	ERAS (*n* = 39)	Control (*n* = 41)	Effect size (95% CI)	*P*
Primary outcomes				
Postoperative LOS, hours/($\bar{x}$ ± s)	112.14 ± 30.33	154.87 ± 33.90	−42.73 (−57.07, −28.39)	<0.001
Total cost of hospitalization, yuan ($\bar{x}$ ± s)	40483.02 ± 5441.85	42942.12 ± 4940.36	−2459.10 (−4770.56, −147.63)	0.037
Secondary outcomes				
30-day readmission [*n* (%)]	0 (0%)	0 (0%)	–	–
30-day reoperation rate [*n* (%)]	0 (0%)	0 (0%)	–	–
Postoperative complications [*n*(%)]				
Fever [*n* (%)]	0 (0%)	1 (2.4%)		1
Acute urine retentions [*n* (%)]	0 (0%)	4 (9.8%)	OR: 0.11 (0.01, 2.06)	0.116
Overall postoperative complication rate [*n* (%)]	0 (0%)	5 (12.2%)	OR: 0.08 (0.004 to 1.41)	0.055
PONV [*n* (%)]	1 (2.6%)	8 (19.5%)	OR:0.11 (0.01 to 0.90)	0.029

### Secondary outcomes measures

Postoperative complication rates are summarized in Table 4. There were no cases of mortality or 30-day readmission in either group. Fever occurred in one patients and acute urinary retention (postvoid residual urine volume >100 mL) was observed in four patients in the control group; neither complication showed a statistically significant difference between groups (*p* > 0.05). All four patients with urinary retention had reported preoperative symptoms of incomplete bladder emptying. These patients underwent recatheterization for 72 h, after which catheter removal resulted in normal voiding with residual volumes below 100 mL. The incidence of postoperative nausea and vomiting (PONV) was significantly lower in the ERAS group (1 case vs. 8 cases in the control group; *p* < 0.05).

No significant differences were observed between the ERAS and control groups in other intraoperative indicators, including fluid administration, blood loss, or operative time (all *p* > 0.05; Table 5). However, the ERAS group showed significantly accelerated recovery across multiple functional metrics, with shorter durations of urinary catheter indwelling, time to first flatus, time to first ambulation, time to full unassisted ambulation, and time to return to basic activities of daily living (ADLs) (all *p* < 0.05; Table 5).

**Table 5:** Key measures on ERAS protocol for pelvic floor reconstruction surgery.

Measures	ERAS (*n* = 39)	Control (*n* = 41)	Effect size (95% CI)	*P*
Intraoperative fluid volume mL/($\bar{x}$ *± s*)	1417.95 ± 494.50	1542.20 ± 586.93	−124.25 (−366.44, 117.94)	0.310
Intraoperative bleeding mL/[M(Q1, Q3)]	100 (40, 200)	100 (50, 200)	–	0.597
Surgery time minutes/($\bar{x}$ ± s)	170.56 ± 86.44	175.78 ± 82.67	−5.22 (−42.86, 32.43)	0.783
Postoperative urinary catheter indwelling time (h), M (Q1, Q3)	23 (21.5, 23.5)	48 (27.25, 70.25)	–	<0.001
Postoperative first exhaust time hours/($\bar{x}$ ± s)	20.10 ± 8.82	26.46 ± 12.76	−6.36 (−11.23, −1.49)	0.011
first postoperative off-bed activity time hours/[M(Q1, Q3)]	24.75 (23,27.5)	44.5 (28, 70)	–	<0.001
Time to full, unassisted ambulation* hours/[M(Q1, Q3)]	28.0 (24.0, 36.0)	52.0 (36.0, 96.0)	–	<0.001
Time to return to basic activities of daily living (ADLs)* days/[M(Q1, Q3)]	3.0 (2.0, 4.0)	7.0 (5.0, 10.0)	–	<0.001
PGI-I score (1–7)			–	1
7 (very favorable)	35	37		
6 (favorable)	4	4		

*Full, unassisted ambulation was defined as independent walking for >20 m without assistance. Return to basic Activities of Daily Living (ADLs) was defined as the ability to independently perform dressing, feeding, grooming, and toileting.

At the 30-day postoperative follow-up, there were no significant differences between the groups in subjective improvement of prolapse symptoms, as assessed by the Patient Global Impression of Improvement (PGI-I) questionnaire, or in objective anatomical restoration based on POP-Q staging (Tables 5, 6). These findings indicate that the ERAS protocol can reduce hospitalization costs and length of stay while improving perioperative comfort, without compromising therapeutic efficacy.

**Table 6:** Comparison of POP-Q scores between the two groups at 30 days after operation.

POP-Q	ERAS (*n* = 39)	Control (*n* = 41)	*P*
Aa	−3.00 (−3.00 ~ −3.00)	−3.00 (−3.00 ~ −3.00)	1.000
Ba	−3.00 (−3.00 ~ −3.00)	−3.00 (−3.00 ~ −3.00)	1.000
C	−6.00 (−6.00 ~ −6.00)	−6.00 (−6.00 ~ −5.75)	0.652
TVL	7.00 (6.13 ~ 7.00)	6.00 (6.00 ~ 7.00)	0.243
Ap	−3.00 (−3.00 ~ −3.00)	−3.00 (−3.00 ~ −3.00)	1.000
Bp	−3.00 (−3.00 ~ −3.00)	−3.00 (−3.00 ~ −3.00)	1.000
D	−7.00 (−7.00 ~ −6.25)	−7.00 (−7.00 ~ −6.00)	0.754

Pain scores assessed at 2 h, 12 h, postoperative day (POD) 1, and POD 2 did not differ significantly between the groups (Table 7). Similarly, quality of recovery, as measured by the QoR-15 questionnaire on POD 3, POD 7, and at 30 days post-surgery, showed no statistically significant differences (Table 8).

**Table 7:** Postoperative pain assessment of two groups of patients (VAS0-10).

Measures	Rest pain assessment		*P*	Movement pain assessment		*P*
	ERAS (*n* = 39)	Control (*n* = 41)		ERAS (*n* = 39)	Control (*n* = 41)	
2 h after operation			0.834			0.865
Mild (VAS 0–3), *n* (%)	36 (92.3%)	38 (92.7%)		34 (87.2%)	36 (87.8%)	
Moderate (VAS 4–6), *n* (%)	3 (7.7%)	2 (4.9%)		5 (12.8%)	4 (9.8%)	
Severe (VAS 7–10), *n* (%)	0 (0%)	1 (2.4%)		0 (0%)	1 (2.4%)	
12 h after operation			1			0.195
Mild (VAS 0–3), *n* (%)	38 (97.4%)	40 (97.6%)		35 (89.7%)	40 (97.6%)	
Moderate (VAS 4–6), *n* (%)	1 (2.6%)	1 (2.4%)		4 (10.3%)	1 (2.4%)	
Severe (VAS 7–10), *n* (%)	0 (0%)	0 (0%)		0 (0%)	0 (0%)	
1 day after operation		–				0.234
Mild (VAS 0–3), *n* (%)	39 (100%)	41 (100%)		37 (94.9%)	41 (100%)	
Moderate (VAS 4–6), *n* (%)	0 (0%)	0 (0%)		2 (5.1%)	0 (0%)	
Severe (VAS 7–10), *n* (%)	0 (0%)	0 (0%)		0 (0%)	0 (0%)	
2 days after operation		–				0.487
Mild (VAS 0–3), *n* (%)	39 (100%)	41 (100%)		38 (97.4%)	41 (100%)	
Moderate (VAS 4–6), *n* (%)	0 (0%)	0 (0%)		1 (2.6%)	0 (0%)	
Severe (VAS 7–10), *n* (%)	0 (0%)	0 (0%)		0 (0%)	0 (0%)	

**Table 8:** QoR-15 scores of patients in the two groups *[M(Q1, Q3)].*

Group/statistic	3 days postoperative (QoR-15)	7 days postoperative (QoR-15)	30 days postoperative (QoR-15)
ERAS (*n* = 39)	142 (135, 143)	143 (141, 143)	144 (142, 144)
Control (*n* = 41)	141 (136.5, 143)	142 (139.5, 143)	143 (143, 144)
*z*	−0.436	−1.22	−1.009
*P*	0.663	0.222	0.313

## Discussion

ERAS protocol is an evidence-based, multimodal perioperative care pathway designed to mitigate surgical stress and promote rapid patient recovery (17). First established in European colorectal surgery in the 1990s, ERAS has been widely adopted across various surgical specialties and is strongly recommended in current clinical guidelines (18–20). The fundamental principle of ERAS involves implementing a series of optimized interventions during the preoperative, intraoperative, and postoperative periods. These measures are systematically applied to alleviate physiological and metabolic stress, maintain normal physiological function, lower complication rates, and ultimately reduce the length of hospitalization (21). A patient-centered approach, emphasizing individualized care within a humanitarian framework, is central to this model (22).

POP is a highly prevalent condition, particularly among older women, for which surgical intervention remains the cornerstone of management in severe cases (23). Pelvic floor reconstruction surgery is a commonly employed procedure; however, it induces considerable physiological stress, often resulting in postoperative pain, catheter-related discomfort, and delayed recovery. Substantial evidence confirms that implementing ERAS protocols in gynecological surgery effectively mitigates the surgical stress response and enhances recovery quality and patient satisfaction (24, 25).

Our findings align with international studies on ERAS in gynecologic surgery, which consistently report shortened hospital stays and reduced complications without compromising safety. For example, a randomized controlled trial involving older patients undergoing transvaginal pelvic floor reconstruction demonstrated that an ERAS protocol significantly decreased postoperative length of stay and pain scores, promoted opioid-sparing analgesia, and lowered the incidence of postoperative nausea and vomiting (26). These advantages are consistent with outcomes observed in broader abdominal and gynecological surgeries, where ERAS has reliably led to shorter hospitalizations and improved patient satisfaction (27, 28). Furthermore, evidence from abdominal wall reconstruction and urogynecological studies supports the efficacy of ERAS in pelvic floor surgery, showing reduced hospitalization duration and opioid consumption without increasing complication or readmission rates (29, 30).

The systematic ERAS approach—integrating preoperative education, multimodal analgesia, and early mobilization—is instrumental in achieving these favorable outcomes. Guided by these principles, we implemented a comprehensive set of ERAS measures tailored to patients undergoing pelvic floor reconstruction for severe POP. Our experience confirms that this structured pathway not only yields satisfactory clinical results but also contributes to standardizing and optimizing perioperative care for this population.

Analysis of recovery indicators showed improved intestinal function recovery in the ERAS group, attributable to several factors: avoidance of mechanical bowel preparation, optimized preoperative fasting, postoperative gum chewing, and early feeding. Early enteral nutrition has been demonstrated to promote gastrointestinal recovery. Although preoperative bowel preparation is commonly used, studies indicate it does not reduce surgical site infections or anastomotic leakage but may cause anxiety, dehydration, and electrolyte imbalances (31). Prolonged fasting can lead to thirst, hunger, and insulin resistance, potentially impairing recovery (32). Our results confirm the safety and feasibility of these preoperative ERAS measures in POP surgery. The lack of significant difference in time to first flatus between groups may be due to individual variations.

ERAS guidelines recommend intraoperative fluid restriction to prevent complications such as pulmonary and gastrointestinal edema, and maintenance of normothermia to prevent stress-related hormonal complications (33). As most POP patients are elderly, particular attention was paid to preventing deep vein thrombosis using elastic stockings. The lower complication rate in the ERAS group may reflect optimized perioperative management, including fluid control, warming, and early oral intake. Although the overall complication rate did not reach statistical significance (0% vs. 12.2%, *p* = 0.055), this clinically relevant reduction suggests a potential safety benefit observed in other ERAS studies (34). The nonsignificant *p*-value may reflect the limited sample size.

Multimodal antiemetic therapy in the ERAS group reduced postoperative nausea and vomiting incidence. Early urinary catheter removal within 24 h did not increase urinary retention rates but facilitated earlier mobilization and improved comfort. Patients encouraged to ambulate on postoperative day 1 experienced shorter hospital stays and enhanced recovery. Consistent discharge criteria confirmed that shorter hospitalization in the ERAS group reflected accelerated recovery. The associated reduction in hospitalization costs alleviated economic burdens and improved healthcare efficiency.

Notably, despite multimodal analgesia in the ERAS group, postoperative pain scores showed no significant intergroup difference. This may be partly explained by the inclusion of middle-aged and elderly patients with potentially reduced pain sensitivity (30), leading to lower baseline scores in both groups. Additionally, it is important to acknowledge that the relatively small sample size may have limited the statistical power of our study, not only constraining the ability to detect a significant difference in pain scores but also potentially masking differences in less frequently occurring outcomes, such as specific complications.

Our study has limitations. Firstly, its retrospective and single-center design introduces the potential for selection and information biases, which may influence the outcomes. The relatively small cohort size from a single tertiary care center limits the generalizability of our findings, as the feasibility and outcomes of ERAS implementation might differ in rural or secondary care settings with varying resources and patient populations. Secondly, the 30-day follow-up period is insufficient to evaluate long-term outcomes critical to POP surgery, such as prolapse recurrence, sustained functional improvement, or mesh-related complications. Future research should also explore potential barriers to widespread ERAS implementation in China, such as institutional protocols, patient education, and resource availability. Larger-scale, multi-center, prospective studies with extended follow-up periods are necessary to validate these findings and assess long-term efficacy. Furthermore, hospitals should develop tailored implementation strategies when adopting ERAS protocols to ensure successful integration into clinical practice.

In summary, ERAS implementation in pelvic floor reconstruction surgery is safe and effective, enhancing recovery, reducing hospital stay and costs, and improving patient comfort without increasing perioperative risks. These findings support integrating ERAS pathways into routine POP surgical care.

## Data Availability

The raw data supporting the conclusions of this article will be made available by the authors, without undue reservation.

## Footnotes

^1^ https://www.chictr.org.cn/
